# The Association between Sex and Risk of Alzheimer’s Disease in Adults with Down Syndrome

**DOI:** 10.3390/jcm10132966

**Published:** 2021-07-01

**Authors:** Pooja Girish Mhatre, Joseph H. Lee, Deborah Pang, Warren B. Zigman, Benjamin Tycko, Sharon J. Krinsky-McHale, Yuchen Yang, Wayne Silverman, Nicole Schupf

**Affiliations:** 1 Department of Epidemiology, School of Public Health, Columbia University, New York, NY 10027, USA; JHL2@cumc.columbia.edu (J.H.L.); ns24@cumc.columbia.edu (N.S.); 2 San Francisco Department of Public Health, San Francisco, CA 94102, USA; 3 Departments of Neurology and Psychiatry, Taub Institute for Research on Alzheimer’s Disease and the Aging Brain, Columbia University, New York, NY 10027, USA; 4 New York State Institute for Basic Research in Developmental Disabilities, Staten Island, NY 10314, USA; Deborah.I.Pang@opwdd.ny.gov (D.P.); WARREN.ZIGMAN@opwdd.ny.gov (W.B.Z.); Sharon.Krinsky-McHale@opwdd.ny.gov (S.J.K.-M.); 5 Hackensack University Medical Center, Hackensack, NJ 07601, USA; benjamin.tycko@hackensackmeridian.org; 6 Department of Biostatistics, Epidemiology and Informatics, University of Pennsylvania, Philadelphia, PA 19104, USA; yangyc1608@gmail.com; 7 Department of Pediatrics, University of California, Irvine, Irvine, CA 92161, USA; wsilverm@hs.uci.edu

**Keywords:** Alzheimer’s Disease, Down Syndrome, sex difference, *APOE4*

## Abstract

Background: Sex differences in the risk of Alzheimer’s Disease (AD) in adults with Down Syndrome (DS) have not been extensively investigated, and existing studies have found conflicting results. This study examined the effect of sex on the risk of AD in adults with DS, adjusted for covariates. Methods: Adults with DS were assessed longitudinally for the development of AD. Competing risk survival analyses were used to determine the effect of sex alone and after adjustment for *APOE-*ε4 status, ethnicity, and level of intellectual disability (ID). Results: Sex differences were significant only in adults over 60 years of age, where men with DS were 6.32 (95% CI: 2.11–18.96, *p* < 0.001) times more likely to develop AD compared with age-matched women with DS. Conclusions: There is an age-associated effect of sex on the risk of AD, with men over 60 years old having six times the risk of AD compared with age-matched women, independent of *APOE*-ε4 status, ethnicity, and level of ID.

## 1. Introduction 

Adults with Down Syndrome (DS) are at high risk of Alzheimer’s Disease (AD), with almost all adults developing AD-associated neuropathology by age 40 years, and the cumulative risk of dementia reaching 50% by their late 50s [1,2]. Currently, people born with DS have a mean life expectancy estimated to be over 60 years of age, a dramatic increase since the first half of the twentieth century, and the population of adults with DS developing AD-related dementia can be expected to grow as their life expectancy continues to increase [3,4,5].

AD causes a form of dementia that progressively destroys memory and other cognitive abilities [6,7]. Neuropathologically, its key characteristics include the deposition of amyloid-beta (Aß) plaques, neurofibrillary tangles, and neurodegeneration [8]. The high risk of dementia in adults with DS has been attributed, at least in part, to the overexpression of the amyloid precursor protein (*APP*) located on chromosome 21, which is triplicated in Down Syndrome [5,9,10]. This overexpression is associated with increased Aß aggregation, the deposition of Aß plaques, and associated cognitive deficits [9,10]. However, there is variability in age at the onset of AD, suggesting the importance of additional risk factors.

There is some evidence that women appear to be at greater risk of developing AD compared with men, independent of the longer life expectancy of women [11,12,13,14,15]. However, there is inconsistency in the literature, with most studies reporting a higher risk in women, some reporting no difference in the risk of AD by sex, and a few studies reporting a higher risk in men [7,16,17,18,19]. Data from the Framingham Study revealed that women experienced a higher lifetime risk of AD compared with age-matched men at age 65 and above [19]. A study examining longitudinal data from cohorts in 12 countries found a faster decline in memory scores for women compared with men and concluded that women experienced a steeper decline in general cognition with increasing age and a higher prevalence of AD compared with men [15]. A meta-analysis of global studies on the prevalence and the incidence of AD found that both were higher among women compared with men, although these differences did not reach statistical significance [17]. In contrast, the Cognitive Function and Ageing Study from the UK examined the incidence rate for AD over a 20-year period and found that men had higher incidence rates of dementia compared with age-matched women initially, but that the incidence rate decreased over time in men but not in women [18]. Despite these differences, female sex continues to be considered a risk factor for AD.

Most studies examining sex differences in the risk of AD in the typically developing population have focused on factors that increase the risk for women, such as the loss of bioavailable estrogen, which is considered to be a protective factor against AD. Estrogen promotes the growth of and prolongs the survival of cholinergic neurons in brain regions serving cognitive functions, increases cholinergic activity, has antioxidant properties, and regulates the metabolism of *APP* to protect against the formation of Aß plaques [20,21,22,23].

However, there is evidence to suggest that men may be underdiagnosed for AD [24]. Murray and colleagues examined over 1600 AD cases from the State of Florida brain bank and found that men were more likely to demonstrate an atypical clinical presentation of AD compared with women and concluded that men have lower reported levels of AD diagnosis due to their atypical clinical presentations [24].

Sex differences in the risk of AD in adults with DS have not been extensively investigated. The few studies that have compared women and men with DS have found conflicting results, with different studies the showing onset for women being earlier [25,26], later [27], or no different from that of men [28,29]. Two studies have employed survival methods to examine age at onset distributions by sex in adults with DS, adjusting for both age and the level of learning disability, and found conflicting results [26,27]. Schupf and colleagues found that men with DS were three times as likely as women to develop AD by age 65 [27]. Both men and women with DS showed elevations of follicle stimulating hormone and luteinizing hormone at puberty, indicative of a primary gonadal dysfunction, which appeared to progress with age and to be more frequent in men than in women [30,31,32,33]. Thus, older men with DS may not benefit from the relative preservation of the hormones proposed to account for lower the risk of AD in men in the general population. ln contrast, Lai and colleagues found that women with DS were approximately twice as likely to develop dementia as men [26].

Studies in the typically developing population have found that older age and the presence of an *APOE*-ε4 allele were the strongest factors which increased the risk of AD [34,35]. The mixed results regarding the impact of sex on AD risk might be associated with an interaction with the apolipoprotein (*APOE*) genotype. *APOE* is a cholesterol carrier that binds to the Aβ peptide and helps clear it from the brain [36]. Of the three polymorphisms in the *APOE* gene, ε4 is said to be the strongest risk factor for late-onset AD [36,37]. Some studies in the general population have demonstrated that the ε4 variant of the *APOE* gene confers a greater risk of AD in women compared with men [14,38,39,40]. Altmann and colleagues examined the medical records of more than 8000 people and found that initially healthy *APOE*-ε4-positive women were twice as likely to develop AD compared with their *APOE*-ε4-negative counterparts, while the risk among *APOE*-ε4-positive and -negative men was nearly equivalent [38]. A study by Neu and colleagues found that men and women with one copy of *APOE*-ε4 had nearly identical odds of developing AD across the span of 55–85 years of age, but between the ages of 65 and 75, women with one copy of the ε4 allele had an increased risk of AD compared to men with one copy [39]. Hobel and colleagues found that while both men and women with an *APOE*-ε4 allele experienced changes in the volumes of the hippocampus and the amygdala, only women experienced a decline in cognition, and women with two *APOE*-ε4 alleles had poorer memory and global cognition compared with their male counterparts who also had two *APOE*-ε4 alleles [40]. 

There are inconsistencies in the literature about the role of *APOE*-ε4 as a risk factor for AD in the population with DS [41]. In numerous studies, the presence of an ε4 allele was associated with a significantly greater risk of, and an earlier age of onset of, AD [5,42,43,44,45]. However, some studies failed to find a significant effect of *APOE*-ε4 on the onset of AD in the population with DS, although the differences that were reported were descriptively adequate in the expected direction of an increased risk with E4 [41,46,47]. Prasher and colleagues conducted a meta-analysis looking at the association between *APOE*-ε4 status and AD onset and did not find a significant association between the two, but reported that adults with DS with the ε4 allele had a tendency towards a lower age of onset of dementia [48]. In addition, Lai and colleagues found no significant interaction between sex and *APOE*-ε4 status regarding the risk of AD in the population with DS [49].

This study followed a cohort of adults with DS evaluated over a period of up to 17 years (mean = 5.7 years, SD = 4.3) and ranging in age from 30 to 78 at baseline. Analyses focused on the effect of sex on the risk of AD in the sample with DS, while adjusting for *APOE* genotypes and other covariates. 

## 2. Materials and Methods

### 2.1. Study Participants

Study participants were 30 years of age and older at the time of enrollment (range: 30–78 years) and resided in New York, Connecticut, New Jersey, or eastern Pennsylvania. Participants were recruited with the help of state and voluntary service provider agencies and were eligible for inclusion in the present study if: (1) a “legally authorized representative (LAR)” provided informed consent; (2) the participant either provided consent or assent indicating willingness to participate; and (3) the participant was willing and able to provide blood samples. Recruitment, informed consent, and study procedures were approved by the Institutional Review Boards of the New York State Institute for Basic Research in Developmental Disabilities, the Columbia University Medical Center, and the Johns Hopkins University School of Medicine. 

### 2.2. Assessments of AD-Dementia Status

Assessments were conducted at the time of study entry and were repeated at intervals of approximately 18 months for up to 5 cycles of follow-up. The mean duration of follow-up was 5.7 years (SD = 4.3). Assessments included evaluations of cognition and functional abilities, behavioral/psychiatric conditions, and an examination of medical records for information on health status and medication usage. Cognitive function was evaluated with a test battery designed for use in individuals experiencing lifelong cognitive impairment but varying widely in their initial levels of intellectual functioning, as previously described [50]. Structured interviews were conducted with caregivers to collect information on adaptive behavior and neuropsychiatric concerns. Past and current records were reviewed for all participants.

### 2.3. Classification of AD-Dementia

After each assessment cycle, a classification of AD status was made during a consensus case conference for each individual, based on the evidence of stability or decline in performance profiles over time [50]. Each individual was classified as: (1) *Cognitively Stable*, indicating with reasonable certainty that significant impairment was absent; (2) *MCI-DS,* indicating that there was evidence of mild cognitive or functional decline, but the observed change did not meet the criteria for AD dementia; (3) *possible AD*, indicating that some signs and symptoms of AD were present but the decline over time was not convincing enough to meet the criteria for probable AD; and (4) *probable AD*, indicating with confidence that AD was present based on substantial decline over an extended period of time. For the analysis purposes in this study, AD status was collapsed into the two categories of cognitively stable, which included non-demented individuals and individuals with MCI, and incident dementia, which included all individuals with possible AD and probable AD. 

### 2.4. Apolipoprotein E Genotypes

The apolipoprotein E (APOE) genotyping employed standard PCR-RFLP methods using the Hha 1 (Cfol) digestion of an *APOE* genomic PCR product spanning the polymorphic (cys/arg) sites at codons 112 and 158. Acrylamide gel electrophoresis was used to assess and document the restriction in fragment size [51]. 

### 2.5. Statistical Analysis

Frequency distributions were used for descriptive analyses, while Student’s *t*-tests and chi-square tests for independent samples were used to examine the age at onset of AD distributions by sex, ethnicity, level of intellectual disability, and *APOE* status. Competing risks survival analysis models were used to assess the influence of sex on the risk of AD. These models have been used in other studies assessing risk factors for AD [52,53]. A competing risk is defined as an outcome that precludes the outcome of interest [54]. For these models, death from AD was the outcome of interest, with non-AD-related death as the competing risk. Each individual was considered to be at risk of AD from age at first visit until current age at the end of the study, age at death (if unaffected), or age at the onset of AD. We compared the cumulative incidence for AD by sex using a non-parametric cumulative incidence function (CIF). Cox regression models with the level of intellectual disability, *APOE*-ε4 allele status, and ethnicity were used as covariates to control for the potential confounding of the association between sex and AD and to evaluate whether these factors were independent risk factors for AD. The competing risks survival analyses accounted for both left truncation, which excludes the prevalent cases of AD at baseline, and right censoring, which excludes cases where the age at AD onset could not be determined in the follow-up time of the study [55].

First, we ran a competing risks Cox regression model comparing the age at onset distributions and the risk of AD by sex, using women as the reference group. We then assessed the relationship, adjusting for *APOE-*ε4 status, ethnicity, and the level of intellectual disability as covariates. We also examined the independent risk of *APOE-*ε4 status on the risk of AD using a competing risks Cox regression model. 

In competing risks survival analyses, it is common to encounter time-dependent covariates, defined as factors that do not remain constant over the course of the study [56]. Since the associations between the covariates and the risk of AD changed over time due to age, we reassessed the relationship between sex and AD using *APOE*-ε4 status, level of intellectual disability, and ethnicity as time-dependent covariates for ages 60 years and under and over 60 years. The hazard function is as follows:

$$\lambda \left(t\right)=\{\begin{array}{c} \lambda_{0}\left(t\right)e^{\left\{\;\beta_{1}sex+\beta_{2}APOE4+\beta_{3}LOF+\beta_{4}Ethnicity\right\}}\;t\leq 60\\ \lambda_{0}\left(t\right)e^{\left\{\;\beta_{5}sex+\beta_{6}APOE4+\beta_{7}LOF+\beta_{8}Ethnicity\right\}}\;t>60 \end{array}$$

All statistical procedures were run using IBM SPSS Version 24 and R (programming language).

## 3. Results

Of the 612 participants recruited for the study, 408 participants were included in the final analysis (66.7% of participants). Participants with missing or unknown values for *APOE* genotype, prevalent dementia, missing or unknown values for level of intellectual disability, and missing or unknown values for dementia diagnosis and AD diagnosis were excluded. A total of 267 women and 141 men were included in the analysis (Table 1). Participants were followed up to 17.4 years (mean = 5.7 years, SD = 4.3). Participants who developed dementia were older at baseline and were more likely to carry an *APOE*-ε4 allele. Men developed dementia at an average age of 57.6 years (S.D. 4.7 years) compared with women, who developed dementia at an average age of 55.4 years (SD 5.5 years) (Table 1). However, men and women did not differ by ethnicity or level of intellectual disability (Table 1). 

**Figure 1:**
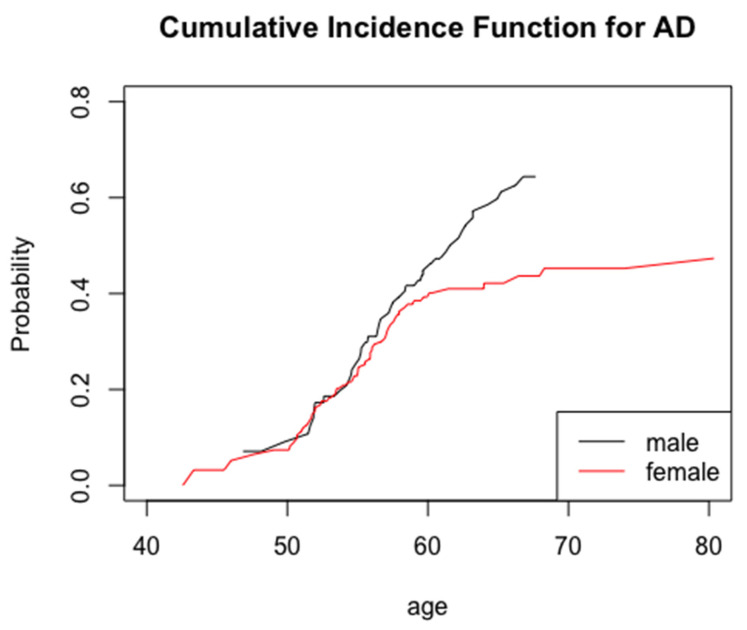
Non-parametric cumulative incidence function for AD separated by sex with left truncation and right censoring.

The cumulative incidence function for AD revealed that a higher percentage of men (~65%) developed AD dementia in this sample compared with women (~45%) (Figure 1). Men and women with DS started to differ in the risk of AD after age 55 (Figure 1). This result was confirmed with the competing risks Cox regression model using time-dependent covariates (Table 2). 

Looking at the effect of sex alone, men with DS had 1.58 times the hazard of AD (95% CI: 1.07–2.36) compared with women with DS (Table 2). After adjusting for the covariates of *APOE*-ε4 status, ethnicity, and level of intellectual disability, men with DS had 1.53 times the hazard of AD (95% CI: 1.03–2.29) compared with women with DS (Table 2). *APOE*-ε4 was also found to be a significant risk factor for AD in this adjusted model, and *APOE*-ε4 allele carriers had 1.79 times the hazard of AD (95% CI: 1.14–2.79) compared with non-carriers (Table 2). The interaction between sex and *APOE*-ε4 status was not statistically significant (hazard ratio: 0.51, 95% CI: 0.21–1.27, *p* = 0.15) and was not included in the final model. 

There was no increase in the hazard of AD associated with sex in participants aged 60 and under. Compared with women, the hazard of AD for men with DS increased by 6.32 times (95% CI: 2.11–18.96) above the age of 60 years (Table 2). Adults with DS aged 60 and under carrying an *APOE*-ε4 allele had nearly twice the hazard of AD compared to adults with DS without an *APOE*-ε4 allele (hazard: 1.93, 95% CI: 1.20–3.08) (Table 2). There was no increase in the hazard of AD for *APOE*-ε4 carriers in the sample with DS over 60 years of age (Table 2). There was no increase in the hazard of AD in the sample with DS by ethnicity or by level of intellectual disability at any age (Table 2). 

## 4. Discussion

The results indicated that men with DS have a greater risk of AD compared to women with DS, but this difference in risk is only apparent over age 60, where men with DS had more than six times the hazard of AD than women with DS (Table 2). 

Our finding that men with DS had a greater risk of AD than women with DS differs from previous studies, which have either found no difference by sex or age or have found that women with DS have a greater risk of AD than men with DS [25,26,28,29,49]. 

One possible reason for these findings is that the lifelong hormonal abnormalities that reduce testosterone conversion to estrogen have a cumulative effect that reaches a critical threshold in old age. Men with DS may therefore have an increased risk of AD in old age compared with women with DS [27]. This possibility is consistent with earlier research, which has demonstrated that men with DS show hormonal abnormalities with testosterone production, and that these abnormalities progress with age [30,31,32,33]. 

It is also possible that an association between multimorbidity with dementia can contribute to the greater risk of AD in men compared to women with DS. In the typically developing population, chronic diseases like diabetes, cardiovascular disease, depression, and inflammatory bowel disease may be associated with an increased risk of AD [35,57,58]. While adults with DS are likely to have unique comorbidity risks, the profiles may also affect AD risk [29,59,60]. The relationship between multimorbidity and dementia in adults with DS has not been extensively investigated. Bayen and colleagues found that adults with DS who developed dementia had a higher frequency of comorbid conditions, suggesting that multimorbidity may contribute to this risk [61]. They also found differences in comorbidities between men and women, suggesting that differential frequencies in these comorbid conditions may confer a greater risk of dementia for men with DS compared to women with DS [61]. 

In the overall sample, the mean age for men was higher at the time of their enrollment compared with women on average, and we considered the possibility that this may have contributed to their increased “over 60” dementia risk. To address this potential confound, we examined dementia status by sex for each cycle of follow-up for an age-matched subsample of men and women with DS, only including individuals above age 60. A greater proportion of men developed MCI/dementia at every follow-up cycle compared with women, so we believe that older age at enrollment did not fully account for the observed increase in dementia risk for older men, as illustrated in Figure 1.

*APOE*-ε4 on its own appeared to confer higher overall risk of AD in adults with DS, but it did not have a differential effect in men compared to women. We suggest that the presence of an *APOE*-ε4 allele in the genotype will accelerate the development of AD and result in an earlier onset, regardless of other factors (sex, ethnicity, and the level of intellectual disability), as it does in the general population (Table 2) [62,63,64]. 

The results of this study indicate that there may be an age-dependent association between sex and the risk of AD in this population. With the inconsistencies in results across prior studies examining the association between sex and AD risk in the population with DS, it will be important to examine sex differences in the mediating effect of other factors, such as comorbidities, metabolites, and genetic markers, on the risk of AD in this population.

## Figures and Tables

**Table 1:** Demographic characteristics by sex and AD dementia status.

	Total Group (408)		Men (141)		Women (267)	
Characteristic	Cognitively Stable	Incident Dementia	Cognitively Stable	Incident Dementia	Cognitively Stable	Incident Dementia
Age at First Visit (mean ± S.D.)	49.2 (6.5)	53.5 (5.2) *	51.8 (4.8)	54.4 (4.7) *	48.0 (6.8)	52.7 (5.5) *
Age at Dementia Onset (mean ± S.D.)	−	56.4 (5.3)	−	57.6 (4.7) *	−	55.4 (5.5) *
Ethnicity (*n*, %)						
Non-White	29 (78.4)	8 (21.6)	5 (62.5)	3 (37.5)	24 (82.8)	5 (17.2)
White	277 (74.7)	94 (25.3)	91 (68.4)	42 (31.6)	186 (78.2)	52 (21.8)
Level of Intellectual Disability (*n*, %)						
Severe/Profound	113 (72.0)	44 (28.0)	34 (65.4)	18 (34.6)	79 (75.2)	26 (24.8)
Mild Moderate	193 (76.9)	58 (23.1)	62 (69.7)	27 (30.3)	131 (80.9)	31 (19.1)
*APOE* Allele (*n*, %)						
ε4 Allele	63 (69.2)	28 (30.8)	19 (65.6)	10 (34.5)	44 (71.0)	18 (29.0)
No ε4 Allele	243 (76.7)	74 (23.3)	77 (68.8)	35 (31.3)	166 (81.0)	39 (19.0)

*p*-Values less than 0.05 are considered to be significant and are marked with *.

**Table 2:** Hazard ratio for AD dementia by sex in adults with Down Syndrome.

Total Group	N	Demented	Hazard Ratio	95% CI
**Sex-Only Model**				
Sex				
Men	141	45 (31.9)	1.58	1.07–2.36 *
Women	267	57 (21.3)	1.0	Reference
***APOE-*** **ε4-Only Model**				
*APOE*				
ε4 Allele	91	28 (27.5)	1.81	1.16–2.82 *
No ε4 Allele	317	74 (20.6)	1.0	Reference
**Full Model**				
Sex				
Men	141	45 (31.9)	1.53	1.03–2.29 *
Women	267	57 (21.3)	1	Reference
*APOE*				
ε4 Allele	91	28 (27.5)	1.79	1.14–2.79 *
No ε4 Allele	317	74 (20.6)	1	Reference
Ethnicity				
Non-White	37	8 (21.6)	0.76	0.36–1.59
White	371	94 (25.3)	1	Reference
Level of Intellectual Disability				
Severe/Profound	157	44 (28.0)	1.12	0.75–1.66
Mild/Moderate	251	58 (23.1)	1	Reference
**≤60 Years**				
Sex				
Men	102	31 (30.4)	1.16	0.74–1.83
Women	206	51 (24.8)	1	Reference
*APOE*				
ε4 Allele	81	26 (32.1)	1.93	1.20–3.08 **
Non-White	29	7 (24.1)	0.79	0.36–1.73
White	279	75 (26.9)	1	Reference
Level of Intellectual Disability				
Severe/Profound	116	35 (30.2)	1.11	0.72–1.73
Mild/Moderate	192	47 (24.5)	1	Reference
Over 60 Years				
Sex				
Men	39	14 (35.9)	6.32	2.11–18.96 ***
Women	61	6 (9.8)	1	Reference
*APOE*				
ε4 Allele	10	2 (20.2)	0.73	0.16–3.33
Ethnicity				
Non-White	8	1 (12.5)	0.78	0.09–6.77
White	92	19 (20.7)	1	Reference
Level of Intellectual Disability				
Severe/Profound	41	9 (22.0)	1.39	0.55–3.50
Mild/Moderate	59	11 (18.6)	1	Reference

*p*-Values less than 0.05 are considered to be significant and are marked with *. *p*-Values less than 0.01 are considered to be significant and are marked with **. *p*-Values less than 0.001 are considered to be highly significant are are marked with ***.
